# Cellular and soluble plasma immune markers at presentation in multisystem inflammatory syndrome in children and Kawasaki disease in South Africa: An observational study

**DOI:** 10.1097/MD.0000000000041516

**Published:** 2025-02-14

**Authors:** Deepthi R. Abraham, Ansia van Coller, Megan M. Tattersall, Edwin Mohlake, Nurea A. Yunis, Kate Webb, Moleen Zunza, Marieke M. van der Zalm, Helena Rabie, Richard H. Glashoff

**Affiliations:** a Department of Pediatrics and Child Health, Tygerberg Academic Hospital and Faculty of Medicine and Health Sciences, Stellenbosch University, Cape Town, South Africa; b Immunology Unit, Division of Medical Microbiology, Department of Pathology, National Health Laboratory Service and Faculty of Medicine and Health Sciences, Stellenbosch University, Cape Town, South Africa; c Genomics Platform, South African Medical Research Council, Cape Town, South Africa; d Division of Paediatric Rheumatology, Department of Paediatrics and Child Health, Red Cross War Memorial Children’s Hospital, University of Cape Town, Cape Town, South Africa; e Division of Epidemiology & Biostatistics, Department of Global Health, Faculty of Medicine and Health Sciences, Stellenbosch University, Cape Town, South Africa; f Department of Paediatrics and Child Health, Desmond Tutu TB Centre, Faculty of Medicine and Health Sciences, Stellenbosch University, Cape Town, South Africa; g Division of Paediatric Infectious Diseases, Department of Paediatrics and Child Health, Faculty of Medicine and Health Sciences, Stellenbosch University, Cape Town, South Africa.

**Keywords:** Inflammatory biomarkers, Kawasaki Disease, MIS-C, SARS-CoV-2

## Abstract

Immune and inflammatory alterations in multisystem inflammatory syndrome in children (MIS-C) as compared to Kawasaki disease (KD) were investigated in South Africa, a region of unique genetic background and high infectious disease burden. The observational study included MIS-C and KD patients during 4 severe acute respiratory syndrome coronavirus 2 waves (June 1, 2020–March 31, 2023) plus 12 healthy controls. Clinical features, routine inflammatory markers, hematological parameters, lymphocyte subsets and plasma inflammatory cytokines/chemokines were compared between groups. We enrolled 68 MIS-C, 18 KD, and 12 healthy controls. MIS-C patients had higher rates of Intensive Care Unit admission compared to KD (46% vs 17%; *P* = .03) and longer hospital stay (8.5 vs 6 days; *P* < .001). 8 MIS-C but no KD patients had an ejection fraction of < 40% (*P* = .07). Median lymphocyte counts were decreased in MIS-C, 1.2 cells/μL (interquartile range 0.7–2.3) versus KD 2.5 cells/μL (interquartile range 1.2–3.7), *P* = .02. Median CD3 + T-cell counts were lower in MIS-C (*P* = .04). Children with MIS-C had a higher median N-terminal pro-B-type natriuretic peptide of 5836 ng/L (1784–25,698) versus 7 ng/L (88–3262), *P* < .001 and Troponin T 25 ng/L (9–73) versus 7 ng/L (4–24), *P* = .01. Majority of cytokines/chemokines were elevated in both MIS-C and KD. When MIS-C was stratified by severity, significant differences in C-reactive protein (*P* < .001), total lymphocytes (*P* = .01), and N-terminal pro-B-type natriuretic peptide (*P* = .01) were observed. Inflammatory cytokine and chemokine levels were markedly raised in both KD and MIS-C. 3 markers were highlighted as indicators of MIS-C severity. There is a strong overlap in inflammatory marker alterations between MIS-C and KD at presentation in the African setting.

## 1. Introduction

Multisystem inflammatory syndrome in children (MIS-C) is a rare, life-threatening post infectious inflammatory condition associated with severe acute respiratory syndrome coronavirus 2 (SARS-CoV-2) infection.^[1]^ MIS-C is characterized by a pathological, delayed, exaggerated and dysregulated innate and adaptive immune response. This dysregulated immune response causes systemic injury and multi-organ dysfunction.^[1,2]^ In children with MIS-C, titers of Immunoglobulin G neutralizing antibodies to SARS-CoV-2 is usually higher than the Immunoglobulin M titer, suggesting a mature adaptive immune response to SARS-CoV-2 including antibody class switching.^[3]^ The clinical features and laboratory characteristics of MIS-C mimic Kawasaki disease (KD) and other febrile illnesses posing a challenge for diagnosis and management.^[4]^ Bacterial and viral infections have been proposed as infectious triggers of KD; however the etiology remains unknown.^[5]^

Children with MIS-C tends to have lower lymphocyte counts, erythrocyte sedimentation rates, albumin and higher transaminitis levels, N-terminal pro-B-type natriuretic peptide (NT-pro BNP), troponin T, C-reactive protein (CRP), D-dimer, fibrinogen, ferritin, and creatinine.^[6–11]^

Elevated interleukin (IL)-8, IL-10 and CXC motif chemokine ligand (CXCL)9 and CXCL10 are reported to be uniquely associated with MIS-C and not KD.^[4,7,10]^ These cytokines and chemokines mediate a broad range of activities including neutrophil recruitment (IL-8), natural killer cell and T-cell recruitment (CXCL9 and CXCL10), and immune regulation/suppression (IL10).^[4,7,10]^ In KD, IL-1β, a nucleotide-binding oligomerization domain-like receptor 3 inflammasome-associated cytokine activates coronary artery endothelial cells resulting in an increased release of IL-6 and IL-8.^[2,3,8,11]^ A strong T helper-17 response that drives IL-17A mediated hyper-inflammation has been reported as distinct to KD.^[2,3,11,12]^

Similar autoantibodies against endothelial cells and antigens of the blood vessels and heart are present in both KD and MIS-C.^[13]^ These autoantibodies may be involved in the activation of the complement system and generation of pro-inflammatory signatures.^[2,3,8,12,14,15]^ Hyper-inflammation and cytokine storms are associated with the up-regulation of endogenous stress signals from damage-associated molecular patterns.^[16]^

Intestinal mucosal dysfunction and epithelial barrier breakdown may support a super-antigen related pathogenesis biological mechanism leading to the development of MIS-C after SARS-CoV-2 infection.^[17,18]^ Gut epithelial damage characterized by elevation of zonulin (a haptoglobin 2 precursor that modulates intestinal epithelial tight junctions) has been noted in MIS-C.^[13,18]^ Gut damage is linked to viral persistence and may provide a portal for viral entry into the bloodstream resulting in an exaggerated chronic systemic immune response.^[18]^

The varied outcomes of inflammatory syndromes such as MIS-C in South Africa are thought to be related to discrepancies within regional health systems and unequal distribution of medical resources.^[19]^ In addition, these children are exposed to a larger burden of infectious diseases from an earlier age with tuberculosis and viruses such as human immunodeficiency virus, Epstein Barr Virus, and Cytomegalovirus.^[20,21]^ South African children have a wide range of ethnic backgrounds and different immune gene signatures.^[22]^ Together this might lead to a different immunological profile and response.

There are limited data from Africa on the inflammatory profiles of children, with MIS-C and KD. A detailed comparison of immune profiles of children with KD and MIS-C sampled over a defined time period during the corona virus disease 2019 (COVID-19) pandemic affords a unique opportunity for improved understanding of both these conditions in South Africa.

The aim of this observational study was to describe the immune and inflammatory marker profiles in MIS-C and KD at presentation and during the acute phase of disease during various SARS-CoV-2 waves of the COVID-19 pandemic in a cohort of children at a tertiary hospital in Cape Town, South Africa.

## 2. Methods

### 2.1. Study period, study design, cohorts and SARS-CoV-2 PCR and antibody testing

Between June 1, 2020 and March 31, 2023, we prospectively enrolled children < 13 years with MIS-C and KD admitted to Tygerberg Hospital in Cape Town, South Africa. Children with suspected MIS-C were enrolled if they met the WHO case definition of MIS-C.^[23]^ Serologic testing for SARS-CoV-2 antibodies became available in August of 2020. Prior to August 2020, children with negative SARS-CoV-2 polymerase chain reaction (PCR) respiratory specimens could enroll if they had contact with a confirmed SARS-CoV-2 case.^[23]^

Children with KD were eligible for enrollment if they met the American Heart Association case definition of KD or atypical KD and had negative testing for respiratory SARS-CoV-2 PCR, Nucleocapsid (N) protein and Spike (S) protein serum antibody to SARS-CoV-2.^[5]^ Children that met the American Heart Association criteria for KD with positive serology or PCR for SARS-CoV-2 were classified as having MIS-C if they met the MIS-C case definition.^[23]^

Between September 2020 and May 2021, a convenience sample of asymptomatic, healthy controls (HC) were recruited at Red Cross War Memorial Children’s Hospital in Cape Town from children presenting for elective surgery.

### 2.2. SARS-CoV-2 waves

The National Institute of Communicable disease Genomic Surveillance reports were used to define the SARS-CoV-2 pandemic waves and the variant of concern associated with each wave. These were as follows: Ancestral type variant from May 3, 2020 to August 16, 2020, Beta variant from November 8, 2020 to February 7, 2021, Delta variant from May 23, 2021 to September 19, 2021 and Omicron variant and subvariants from November 2021 to present.^[24]^

### 2.3. Demographic data and clinical characteristics

Demographic data, clinical characteristics and laboratory results were collected from the medical files as well as the National Health Laboratory Services results database.

Patients were classified as having Severe MIS-C if one or more of the following characteristics were present, the need for: inotropic support, noninvasive and invasive ventilatory support, renal replacement therapy and/or if an ejection fraction of < 40% was recorded. All others were considered “less” severe MIS-C.

### 2.4. Sampling, routine and specialized inflammatory markers

Within 3 days of admission and prior to initiating immune modulatory therapy we collected samples for full blood count with differential count, serum-albumin, CRP, ferritin, NT-pro BNP, troponin T, D-dimer and fibrinogen levels, total CD3 + T cells, CD4 + T cells, CD8 + T-cells, CD19 + B-cells, and CD56 + natural killer-cells as well as CCL7 (MCP-3), CD40L, CXCL10, Fms-like tyrosine-3L, granulocyte macrophage colony stimulating factor, interferon (IFN)-α, IFN‑γ, IL‑1β, IL‑2, IL‑6, IL‑8, IL‑10, IL‑12p70, IL-17A, and tumor necrosis factor-α. We reviewed all available routinely collected results. Lymphocytes were enumerated using BD Multiset^®^ reagents with TruCount^®^ tubes and analyzed using a BD FACSLyric flow cytometer through the National Health Laboratory Services in real time. Plasma samples were immediately stored at −80 °C until analysis. Multiplex Luminex^®^ Discovery Assay (R&D Systems, Minneapolis, MN – LXSAHM-15) was used to assess the plasma concentrations of cytokines and chemokines. Cytokine quantification was performed on cryopreserved plasma samples as per manufacturer’s instructions. The Bio-Plex™ 200 analyzer and Bio-Plex™ manager 6.1.1 software (Bio-Rad Laboratories, Hercules, CA) was used to read the Luminex^®^ plate and collate data. Human S100A8/S100A9 (Calprotectin) Heterodimer Immunoassay and human haptoglobin (HP) Immunoassay commercial enzyme-linked immunosorbent assay kits (R&D Systems, Minneapolis, MN) were used to assess the levels of calprotectin and HP in cryopreserved plasma samples. The manufacturer’s recommended protocol was followed for each enzyme linked immunosorbent assay and the plates were read using the Bio-Rad iMark™ Microplate Reader and Microplate manager software (Bio-Rad Laboratories, Hercules, CA).

### 2.5. Statistical analysis

All data was captured and stored in an anonymized encrypted Excel database. Statistics and data software v17 and GraphPad Prism v10.0.3 (©GraphPad Software, Boston, MA) were used for statistical analysis. Median and interquartile range (IQR) measures were used to describe the continuous data; numbers and proportions were used for dichotomous data. Mann–Whitney tests were used to compare the 2 patient groups’ routine and clinical data depending on data features. analysis of variance and Kruskal–Wallis (nonparametric), with Dunn’s multiple group comparison, with correction for multiple testing, was performed to assess differences in biomarker expression between the 3 study groups (MIS-C, KD, and HC). Spearman correlation tests were performed to determine whether there were any significant relationships between the markers assessed in this study. We tested association between categorical variables using chi-squared test. Statistical significance was set at *P* < .05. The study was exploratory, and no sample size was estimated. Markers with missing data points (i.e., test was not performed) were excluded from analysis.

### 2.6. Ethics

The Health Research Ethics Committees of Stellenbosch University (N20/04/013_COVID-019; N20/07/041, PhD S22/11/240), University of Cape Town (112/2012,599/2020), and Red Cross War Memorial Children’s Hospital (RCC240/WC_202008_113) approved the study. All children and caregivers gave informed consent and/or assent before study procedures were done.

## 3. Results

We recruited 68 children with MIS-C and 18 children with KD at Tygerberg Hospital and 12 HC at Red Cross War Memorial Children’s Hospital (Table 1). 12 of 68 MIS-C patients (18%) fulfilled the KD case definition but had evidence of SARS-CoV‑2 infection and were therefore classified as MIS-C. 49 of 68 (72%) had a positive SARS-CoV-2 serology test and 13/68 (19%) tested SARS-CoV-2 PCR positive at the time of diagnosis. 11 of 19 (58%) had a reported SARS-CoV-2 contact without positive PCR or available serology in the ancestral (1st) COVID-19 wave. There were 15/18 (83%) patients with complete KD and 3/18 (17%) patients with incomplete KD.

**Table 1:** Clinical characteristics and routine laboratory markers in MIS-C and KD patients.

	Normal ranges (NHLS)	Healthy Controls, Median (IQR) n = 12	MIS-C Median (IQR) n = 68	KD Median (IQR) n = 18	MIS-C vs KD
Age (years)	N/A	3.4 (2.5–5.8)	6.2 (2.4–9.2)	2.5 (0.3–3.8)	***P* < .001**
Sex, (%) Male Female	N/A	8 (67%) 4 (33%)	37 (54%) 31 (46%)	13 (72%) 5 (28%)	ns, *P* = .19
Ethnicity, n (%) Black Mixed ancestry	N/A	6 (50%) 6 (50%)	33 (49%) 35 (51%)	13 (72%) 5 (28%)	ns, *P* = .11
Evidence of COVID-19 exposure: n, (%) SARS-CoV-2 serology (positive) Spike (S) Nucleocapsid (N) Serology not available Positive PCR SARS-CoV-2 at diagnosis	N/A	N/A 0 (0%)	49 (72%) 7 (14%) 42 (86%) 17 (25%) 13 (19%)	0 (0%) 0 (0%)	N/A
Duration of symptoms prior to admission; median (IQR)	N/A	0 (0%)	5 (4–7.25)	6.5 (4.25–8)	ns, *P* = .56
All Comorbidities, n (%)	N/A	0 (0%)	10/68 (15%)	5/18 (28%)	
Hospitalization Duration of stay (days) PICU Admission, n (%) Discharged alive, n (%)	N/A N/A N/A	Admission for elective surgery	8.5 (6–11) 31 (46%) 68 (100%)	6 (4.5–6.5) 3 (17%) 18 (100%)	***P* < .001** ***P* = .03**
Number of MIS-C cases fulfilling KD criteria	N/A	None	12 (18%)		
Waves and Variants of Concern: MIS-C and KD cases admitted at each wave 1st wave – Ancestral Type 2nd Wave – Beta Type 3rd Wave – Delta Type 4th Wave – Omicron and Subvariants	N/A	Samples collected between Sept 2020 and May 2021 (Beta wave)	19 (28%) 7 (10%) 27 (40%) 15 (22%)	3 (17%) 5 (28%) 5 (28%) 5 (28%)	
KD Subtypes Complete KD cases Incomplete KD Cases	N/A	N/A	N/A	15 (83%) 3 (17%)	
Kobayashi Score	N/A	N/A	N/A	4.5 (3–5)	
Clinical Symptoms: n (%) Fever Rash Diarrhoea Abdominal pain Conjunctivitis Tachycardia	N/A	N/A	68 (100%) 50 (74%) 34 (50%) 40 (59%) 52 (76%) 68 (100%)	18 (100%) 16 (89%) 12 (67%) 13 (72%) 14 (78%) 16 (89%)	
Interventions (reflecting severity): Requiring Inotropic Support Low Flow and Masked Oxygen Ventilation Renal replacement therapy	N/A	N/A	20 (29%) 19 (28%) 11 (16%) 0 (0%)	3 (17%) 6 (33%) 2 (11%) 0 (0%)	ns, *P* = .19 ns, *P* = .65 ns, *P* = .59
Ejection Fraction (EF) *EF < 40 (Severe cardiac dysfunction)	N/A	N/A	59 (50–64) 8 (12%)	61 (59–71) 0	ns, *P* = .07
Echo Abnormalities, n (%)	N/A	N/A	42 (62%)	9 (50%)	ns; *P* = .37
Coronary Artery Aneurysm	N/A	N/A	7 (10%)	1 (6%)	
CRP	<10 mg/L	N/A	220 (124–307)	204 (97–243)	ns, *P* = .11
Haemoglobin	11.8–14.6 g/dL	N/A	9.7 (8.4–11.0)	9.5 (8.8–10.8)	ns, *P* = .58
Platelet	180–440 × 10^9^/L	N/A	191 (138–325)	251 (91–361)	ns, *P* = .48
Troponin T	≤14 ng/L	N/A	25 (9–73)	7 (4–24)	*P = .01
NT-pro BNP	<450 ng/L	N/A	5836 (1784–25,698)	867 (88–3262)	*P < .001
Ferritin	7–84 ng/L	N/A	605 (304–1277)	308 (205–743.5)	ns, *P* = .07
D-dimer	0.00–0.25 mg/L	N/A	2.2 (1.3–4.2)	1.6 (1.0–4.3)	ns, *P* = .47
Fibrinogen	2.0–4.0 g/L	N/A	5.2 (3.9–6.5)	4.5 (3.5–5.1)	ns, *P* = .30
Albumin	29–42 g/L	N/A	28 (25–32)	29 (22–34)	ns, *P* = 1.00
White cell count	3.90–10.20 × 10^9^/L	N/A	15 (11–23)	18 (8–24)	ns, *P* = .84
Neutrophils	1.7–5.0 × 10^9^/L	N/A	11 (7–17)	11 (6–17)	ns, *P* = .94
Total lymphocytes	1.90–4.30 × 10^9^/L	N/A	1.2 (0.7–2.3)	2.5 (1.2–3.7)	*P = .02
CD3	1200–2600 cells/μL	N/A	868 (644–1703)	1985 (1179–4121)	*P = .04
CD4	4 650–1500 cells/μL	N/A	559 (382–1106)	955 (614–1305)	ns, *P* = .18
CD8	370–1100 cells/μL	N/A	354 (106–704)	850 (202–1099)	ns, *P* = .10
CD19	270–860 cells/μL	N/A	531 (378–1046)	542 (425–2961)	ns, *P* = .37
NK cells	100–480 cells/μL	N/A	92 (46–215)	194 (75–503)	ns, *P* = .09

National Health Laboratory Service (NHLS) standard healthy control ranges were included. Significant differences between MIS-C and KD are indicated in bold. Inflammatory markers associated with MIS-C severity have been indicated with an asterisk (*).

COVID-19 = corona virus disease 2019, CRP = C-reactive protein, EF = ejection fraction, IQR = interquartile range, KD = Kawasaki disease, MIS-C = multisystem inflammatory syndrome in children, n = number of data points, N/A = not applicable, NK = natural killer, ns = not significant, NT-pro BNP = N-terminal pro-hormone of brain natriuretic peptide, PCR = polymerase chain reaction, SARS-CoV-2 = severe acute respiratory syndrome coronavirus 2.

### 3.1. Demographic data

The median age of children with MIS-C was 6.2 (IQR 2.4–9.2) years and they were significantly older than children with KD who had a median age of 2.5 (IQR 0.3–3.8) and HC with a median age of 3.4 (IQR 2.5–5.8) (*P* < .001) (Table 1). More children with KD were male (13/18, 72%) when compared to HC (8/12, 67%) and MIS‑C (37/68, 54%), although this difference was not significant (*P* = .19). There was no difference in the ethnic distribution between the children (*P* = .11).

### 3.2. Clinical characteristics and early outcomes

The duration of symptoms prior to hospitalization was 5 days (IQR 4–7.25) for children with MIS-C and 6.5 days (IQR 4.25–8) for those with KD (*P* = .56). Children with MIS-C had a higher rate of Intensive Care Unit admission (46% vs 17%; *P* = .03) and longer median hospital stay (8.5 vs 6 days; *P* < .001). 8 (12%) children with MIS-C had an ejection fraction of < 40% compared to no children with KD (*P* = .07). There were no deaths in either the MIS-C or KD groups (Table 1).

### 3.3. Routine inflammatory markers, immune cell subsets, and cardiac markers

**Figure 1.**
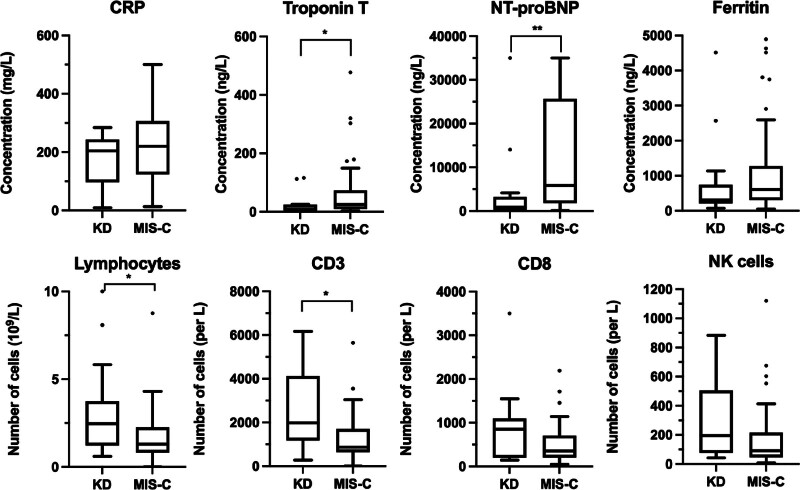
Comparison of CRP, cardiac markers, ferritin and lymphocyte subsets in KD versus MIS-C. Tukey box-and-whisker plots were utilized to visualize the data. Outliers, determined by the Tukey method, are shown in dots. Significant differences between the groups are denoted with *(*P* < .05) and **(*P* < .01). CRP = C-reactive protein, KD = Kawasaki disease, MIS-C = multisystem inflammatory syndrome in children.

Children with MIS-C had lower median total lymphocyte counts 1.2 × 10^9^/L (IQR 0.7–2.3) when compared to KD 2.5 cells/μL (IQR 1.2–3.7); *P* = .02. The median absolute count of CD3 + T cells were lower in MIS-C compared to KD (*P* = .04). Children with MIS-C had a higher median maximum NT-pro BNP of 5836 ng/L (1784–25,698) versus 7 ng/L (88–3262) in KD (*P* < .001) and Troponin T 25 ng/L (9–73) in MIS-C versus 7 ng/L (4–24) in KD (*P* = .01). Although children with MIS-C tended to have higher serum ferritin concentration the difference when compared to KD was not significant 605 ng/L (304–1277) versus 308 ng/L (205–743.5); *P* = .07. There were no significant differences in the levels of CRP, D-dimers, fibrinogen, or albumin between groups (Fig. 1).

### 3.4. Relationship between routine inflammatory markers versus SARS-CoV-2 waves

No significant differences in all laboratory markers tested were observed across the 4 different COVID-19 variant waves (see Fig. S1, Supplemental Digital Content, http://links.lww.com/MD/O369).

### 3.5. Relationship between routine inflammatory markers and severity of disease

The subgroup of MIS-C patients classified with a more severe phenotype (based on cardiac injury and supportive intervention) had higher CRP (*P* < .001) and NT-pro BNP (*P* = .01), and lower lymphocytes (*P* = .01) than children with a less severe MIS-C phenotype (see Fig. S2, Supplemental Digital Content, http://links.lww.com/MD/O369).

### 3.6. Inflammatory cytokines, haptoglobin, and calprotectin

**Figure 2.**
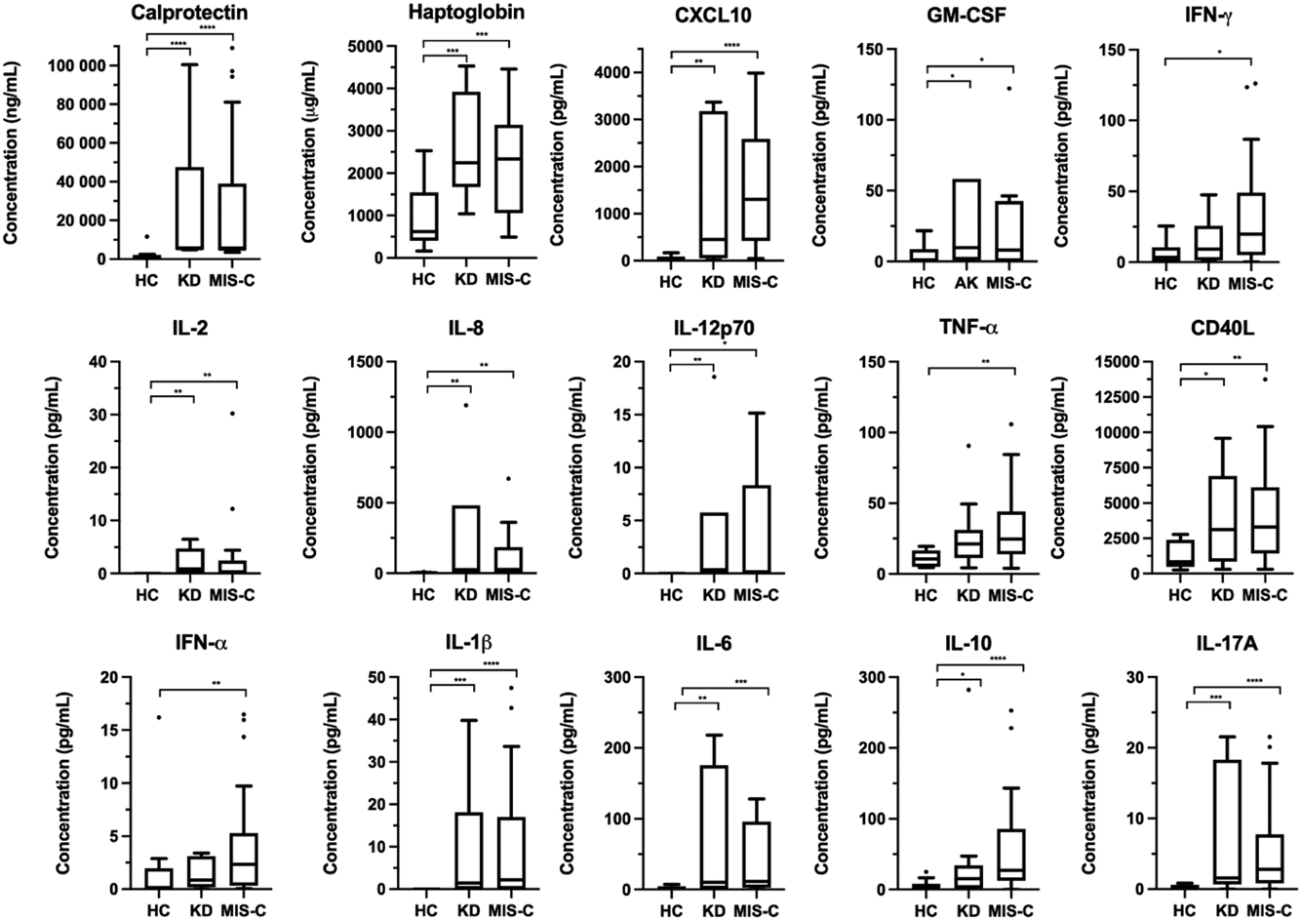
Comparison of gut integrity and inflammatory (cytokine/chemokine) biomarkers for healthy controls (HC), KD, and MIS-C. Tukey box-and-whisker plots were utilized to visualize the data. Outliers, determined by the Tukey method, are shown in dots. Significant differences between the groups are denoted with *(*P* < .05), **(*P* < .01), and ***(*P* < .001). KD = Kawasaki disease, MIS-C = multisystem inflammatory syndrome in children.

Plasma samples were used for quantification of cytokines/chemokines, haptoglobin, and calprotectin in 12 HC, 28 MIS-C, and 10 KD patients (Fig. 2). Calprotectin, HP, CXCL10, granulocyte macrophage colony stimulating factor, IL-2, IL-8, CD40L, IL-1β, IL-6, IL-10, and IL-17A were elevated in KD and MIS-C compared to HC but there was no difference between MIS-C and KD. Elevation in CXCL10, IFN-α, IFN-γ, and IL-10 in MIS-C (vs HC) was noticeably greater than what was seen in KD, even though statistical significance was not attained. IFN-α and IFN-γ levels differed significantly between HC and MIS‑C but not HC and KD (Table 2).

**Table 2:** Inflammatory markers assessed in blood plasma of healthy controls, MIS-C and KD patients.

	HC Median (IQR) n = 12	MIS-C Median (IQR) n = 28	KD Median (IQR) n = 10	ANOVA/ Kruskal–Wallis	HC vs MIS-C	HC vs KD	MIS-C vs KD
Calprotectin (ng/mL)	789 (461–2142)	5099 (4809–39,026)	5368 (4894–47,555)	***P* < .001**	***P* < .001**	***P* < .001**	ns
Haptoglobin (μg/mL)	621 (405.88–1549.15)	2333.78 (1055.54–3140.59)	2247.61 (1670.90–3922.34)	***P* < .001**	***P* < .001**	***P* < .001**	ns
CCL7 (pg/mL)	0 (0–0)	0 (0–4.6)	0 (0–5.4)	ns	ns	ns	ns
CXCL10 (pg/mL)	34 (25–87)	1301 (423–2591)	450 (59–3183)	***P* < .001**	***P* < .001**	***P* = .02**	ns
GM-CSF (pg/mL)	0 (0–8.7)	8.12 (0–42.5)	9.8 (0.9–58.3)	**ns**	ns	ns	ns
IFN-γ (pg/mL)	3.4 (0–10.4)	19.9 (5.1–49.1)	9.1 (1.1–25.6)	***P* = .03**	***P* = .03**	ns	ns
IL-2 (pg/mL)	0 (0–0)	0.3 (0–2.4)	0.9 (0–4.8)	***P* = .01**	***P* = .01**	***P* = .02**	ns
IL-8 (pg/mL)	3.3 (2.2–4.7)	27.3 (4.4–185.9)	26.5 (6.9–480)	***P* = .01**	***P* = .01**	***P* = .02**	ns
IL-12p70 (pg/mL)	0 (0–0)	0 (0–8.4)	0.4 (0–5.8)	***P* = .03**	***P* = .04**	ns	ns
TNF-α (pg/mL)	10.5 (5.2–16.5)	24.6 (13.9–44.3)	21.2 (11.3–31.3)	***P* = .01**	***P* = .01**	ns	ns
CD40L (pg/mL)	836 (496–2380)	3290 (1433–6100)	3114 (860–6913)	***P* = .02**	***P* = .01**	ns	ns
FLT3L (pg/mL)	51.2 (48.6–61.5)	61.4 (17.4–111.1)	63.4 (38.3–96.2)	ns	ns	ns	ns
IFN-α (pg/mL)	0 (0–1.98)	2.3 (0.3–5.3)	0.8 (0.2–3.1)	***P* = .01**	***P* = .01**	ns	ns
IL-1β (pg/mL)	0 (0–0)	2.2 (0–17)	1.4 (0–18.1)	***P* = .01**	***P* < .001**	***P* = .01**	ns
IL-6 (pg/mL)	0 (0–4.5)	11.4 (2.7–95.9)	10.0 (1.2–175.5)	***P* < .001**	***P* < .001**	***P* = .09**	ns
IL-10 (pg/mL)	0.8 (0–8.1)	27.1 (12.5–85.4)	15.3 (1.7–34.2)	***P* < .001**	***P* < .001**	ns	ns
IL-17A (pg/mL)	0 (0–0.6)	2.8 (0.8–7.7)	1.6 (0.7–18.3)	***P* < .001**	***P* < .001**	***P* = .01**	ns

ANOVA/Kruskal–Wallis and multiple comparison statistical testing results (with correction for multiple testing) are indicated in bold.

ANOVA = analysis of variance, CXCL = CXC motif chemokine ligand, FLT3L = Fms-like tyrosine-3L, GM-CSF = granulocyte macrophage colony stimulating factor, HC = healthy control, IFN = interferon, IL = interleukin, IQR = interquartile range, KD = Kawasaki disease, MIS-C = multisystem inflammatory syndrome in children, ns = not significant, TNF = tumor necrosis factor.

## 4. Discussion

This study presents novel data from a South African setting with patients possessing a unique genetic background and high infectious disease burden. We found that inflammatory cytokine and chemokine levels were markedly raised in both KD and MIS-C as compared to healthy children but none of the markers were able to clearly distinguish between these diseases or indeed severity. Our study does, however, highlight 3 markers (CRP, NT-pro BNP, and total lymphocytes) that differentiate severe MIS-C from less severe MIS-C. Immediate outcomes of both conditions in our setting, in general, appear not to be impacted by immune profiles but warrants further investigation. Raised cardiac inflammatory markers helped caution for longer follow-ups.

Despite several reports on inflammatory profiles of children with MIS-C and comparing children with MIS-C and KD, this study is one of few reporting from Africa.^[19,25]^ We found that South African children with MIS-C and KD have similar inflammatory profiles to those in Europe and North America.^[2,4–12]^

The varied outcomes of MIS-C within regions in South Africa may have been impacted by discrepancies within regional health systems.^[19]^ In addition, social determinants of health, unequal distribution of health resources, delayed access to care and limited reporting may influence our understanding of the disease severity and outcomes in Africa.^[19]^ Of particular interest has been the immune profiling status and impact thereof on outcomes.

Most studies compare and stratify inflammatory markers for severity between severe acute COVID-19 and MIS-C or between MIS-C and KD and less so for stratification of severity of MIS-C itself.^[2,4,13,26,27]^ In our study, children satisfying our definition of more severe MIS-C had higher CRP, NT-pro BNP, and lower lymphocytes, when compared with children with less severe MIS-C. The markers are suggestive of a hyper-inflammatory immune response with marked myocardial damage.^[28]^ As seen in other reports children, with MIS-C had more cardiac injury and inflammation that those with KD. Their Troponin T and NT-pro BNP were significantly raised, whereas these indices increased only moderately in children with KD. Similar to other reports seen globally, we found that children with MIS-C have more cardiac inflammation and injury, than most children with KD. The long-term cardiac outcomes are still not clear.^[4,7,28–30]^

Compared to healthy children we found a significant elevation of IFN-α, IL-10, and IFN-γ in children with MIS-C but not in children with KD. In children with MIS-C these markers and CXCL10 were at least double the level observed in children with KD. These cytokines, coupled with the decrease of natural killer cells, selective depletion of T lymphocytes (CD3+) and the CD8 + cytotoxic T cell subset specifically, suggests the critical role of components of innate and adaptive cellular control of active or recent viral infection.^[2,4,6,7,31,32]^ The absence of these changes in KD seems to indicate an alternative or less recent infectious etiology.^[33,34]^

CD3 + T cells and platelets were lower in MIS-C compared to KD suggesting possible cellular trafficking, retention and/or clustering in sites or foci of severe inflammation and/or immune activity.^[13,35]^ In the case of MIS-C the postulated remote site of infection is gut epithelial cells.^[13,18]^ Infection of or viral shedding from gut epithelia may account for hyper inflammation as it triggers restimulation of a recently activated immune system responding to the initial SARS-CoV-2 infection.^[13,18]^

Assessment of inflammatory phagocyte (macrophage and neutrophil) and gut-associated inflammation in our study was focused on calprotectin and HP. Both calprotectin and haptoglobin (related to gut inflammation and damage) were elevated in both diseases as reported elsewhere.^[36,37]^ Calprotectin (S100A8/A9) is produced by reactive cells in an active inflammatory setting, including as previously reported in MIS-C, KD, but also severe COVID-19 and inflammatory bowel disease.^[36–38]^ HP is a hemoglobin-binding, immune-modulatory protein that regulates the immune response and reduces oxidative stress.^[39]^ Zonulin is an uncleaved form of mature pre-haptoglobin-2.^[40]^ These proteins are markers of gastrointestinal gap junction impairment.^[13,17]^ Zonulin is selectively upregulated in MIS-C.^[18,41]^ Accurate assessment of zonulin with the standardized commercial immunoassay testing is problematic; as kits have been reported to not always accurately measure zonulin per se.^[42]^ The increases in calprotectin and HP suggest possible gut barrier integrity loss (HP) involvement. Both processes are operative in MIS-C and KD.

IL-17 cytokines, in particular IL-17A has previously been reported to distinguish KD from other pediatric inflammatory conditions.^[4,7,43]^ However, no significant difference was seen in our comparison of MIS-C and KD. Levels were raised in both MIS-C and KD compared to HC. IL-17A, a pivotal role player in Th17 pro-inflammatory responses, promotes neutrophil function and gut integrity and also contributes to cytokine storm.^[43]^ The similar levels of IL-17A may be reflective of the elevated levels of calprotectin and haptoglobin in both conditions, which in turn appear to implicate gut barrier integrity related pathology. Our findings suggest the gut barrier as an important factor. It may serve as a potential portal for viral (and also possibly gut microbiome constituent) entry into the bloodstream resulting in persistent and exaggerated chronic systemic immune responses in both MIS-C and KD.^[18]^ The similarity in IL-17A levels between MIS-C and KD does appear unique to our study and requires more in-depth investigation.

We previously reported that the clinical and disease course of MIS-C did not change markedly with each new variant-driven wave and that there was a reduction in the median age of children presenting with MIS-C in each of the 4 consecutive waves in South Africa.^[44]^ In this study we showed that inflammatory and immune markers were also similar between waves.

Our study has a number of limitations. We may have misclassified some children with typical KD who had positive serology or PCR of SARS-CoV-2 as MIS‑C. Furthermore, our study has a limited sample size, and the MIS-C and KD groups were imbalanced but represent the incidence of these illnesses seen during the study period.

### 4.1. Conclusion

As seen elsewhere, MIS-C and KD in South African children undoubtedly share features of profound immune responsiveness and inflammation. The distinct differences between them may require further validation through studies applying more advanced analysis including proteomic and transcriptomic analysis. An in-depth longitudinal analysis incorporating clinical and laboratory markers during the recovery phases of the disease are imperative to ascertain whether any of the abnormalities highlighted persist in either condition. This includes the need for prolonged clinical follow-up of cardiac abnormalities combined with any evidence of persisting inflammatory marker abnormalities. More detailed evaluation of gut damage by including additional markers such as intestinal fatty acid binding protein or lipopolysaccharide binding protein and an in-depth probing for evidence of plasma viral components would clarify some of the features highlighted in this study which point towards active viral-directed innate and adaptive cellular immunity in MIS-C.

### 4.2. Clinical significance

This Africa-based study highlights the strong overlap in immune and inflammatory markers between MIS-C and KD over all 4 SARS-CoV-2 variant of concern driven waves. Children with more severe MIS-C had significantly higher CRP and NT-pro BNP and lower lymphocytes when compared with children with less severe MIS‑C.

## Acknowledgments

We acknowledge Ms. Sakenah Larney and the personnel of the National Health Laboratory Service and Stellenbosch University Central Analytical Facility for sample collection, dissemination and storage, and Ms. Candice Snyders for her assistance with the Luminex assays. We acknowledge Ms. Sandile Ndlovu for her expertise and knowledge in helping analyze, illustrate and reflect on some of the statistical models used in the study. Permission was obtained from all the above-mentioned personnel to be named and acknowledged in the manuscript.

## Author contributions

**Conceptualization:** Deepthi R. Abraham, Ansia van Coller, Kate Webb, Marieke M. van der Zalm, Helena Rabie, Richard H. Glashoff.

**Data curation:** Deepthi R. Abraham, Ansia van Coller, Nurea A. Yunis, Kate Webb, Richard H. Glashoff.

**Formal analysis:** Ansia van Coller, Moleen Zunza.

**Funding acquisition:** Deepthi R. Abraham, Richard H. Glashoff.

**Investigation:** Deepthi R. Abraham, Ansia van Coller, Richard H. Glashoff.

**Methodology:** Deepthi R. Abraham, Ansia van Coller, Megan M. Tattersall, Edwin Mohlake, Richard H. Glashoff.

**Project administration:** Deepthi R. Abraham, Richard H. Glashoff.

**Resources:** Deepthi R. Abraham, Ansia van Coller, Kate Webb, Marieke M. van der Zalm, Helena Rabie, Richard H. Glashoff.

**Software:** Ansia van Coller, Moleen Zunza.

**Supervision:** Marieke M. van der Zalm, Helena Rabie, Richard H. Glashoff.

**Validation:** Ansia van Coller.

**Visualization:** Ansia van Coller.

**Writing – original draft:** Deepthi R. Abraham, Ansia van Coller, Richard H. Glashoff.

**Writing – review & editing:** Deepthi R. Abraham, Ansia van Coller, Megan M. Tattersall, Edwin Mohlake, Nurea A. Yunis, Kate Webb, Moleen Zunza, Marieke M. van der Zalm, Helena Rabie, Richard H. Glashoff.

## Supplementary Material


