# Supplementary material for: Cellular and soluble plasma immune markers at presentation in multisystem inflammatory syndrome in children and Kawasaki disease in South Africa: An observational study
Source: Medicine (Baltimore). 2025 Feb 14;104(7):e41516. doi: 10.1097/MD.0000000000041516 (PMC11835083; doi:10.1097/MD.0000000000041516)

Supplementary Figure 1: Comparison of biomarkers for MIS-C across different COVID-19 waves. Tukey box-and-whisker plots were utilized to visualize the data. Outliers, determined by the Tukey method, are shown in dots. There was no biomarker data points for wave 1A. 1A = Ancestral variant; 2B = Beta variant; 3D = Delta variant; 4O = Omicron variant.

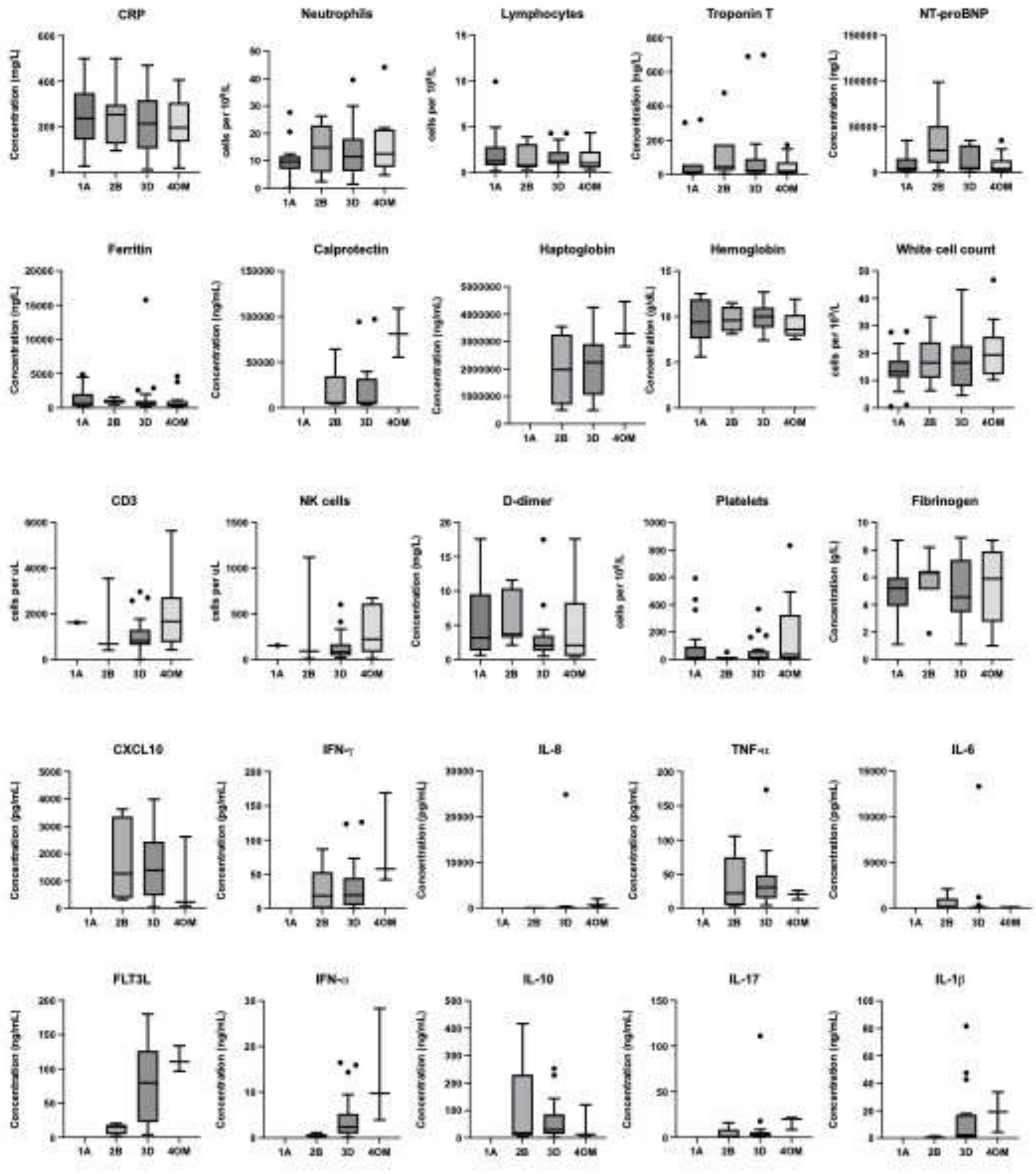

Supplementary Figure 2: Comparison of biomarkers for severe vs less-severe MIS-C and KD cases. Tukey box-and-whisker plots were utilized to visualize the data. Outliers, determined by the Tukey method, are shown in dots.

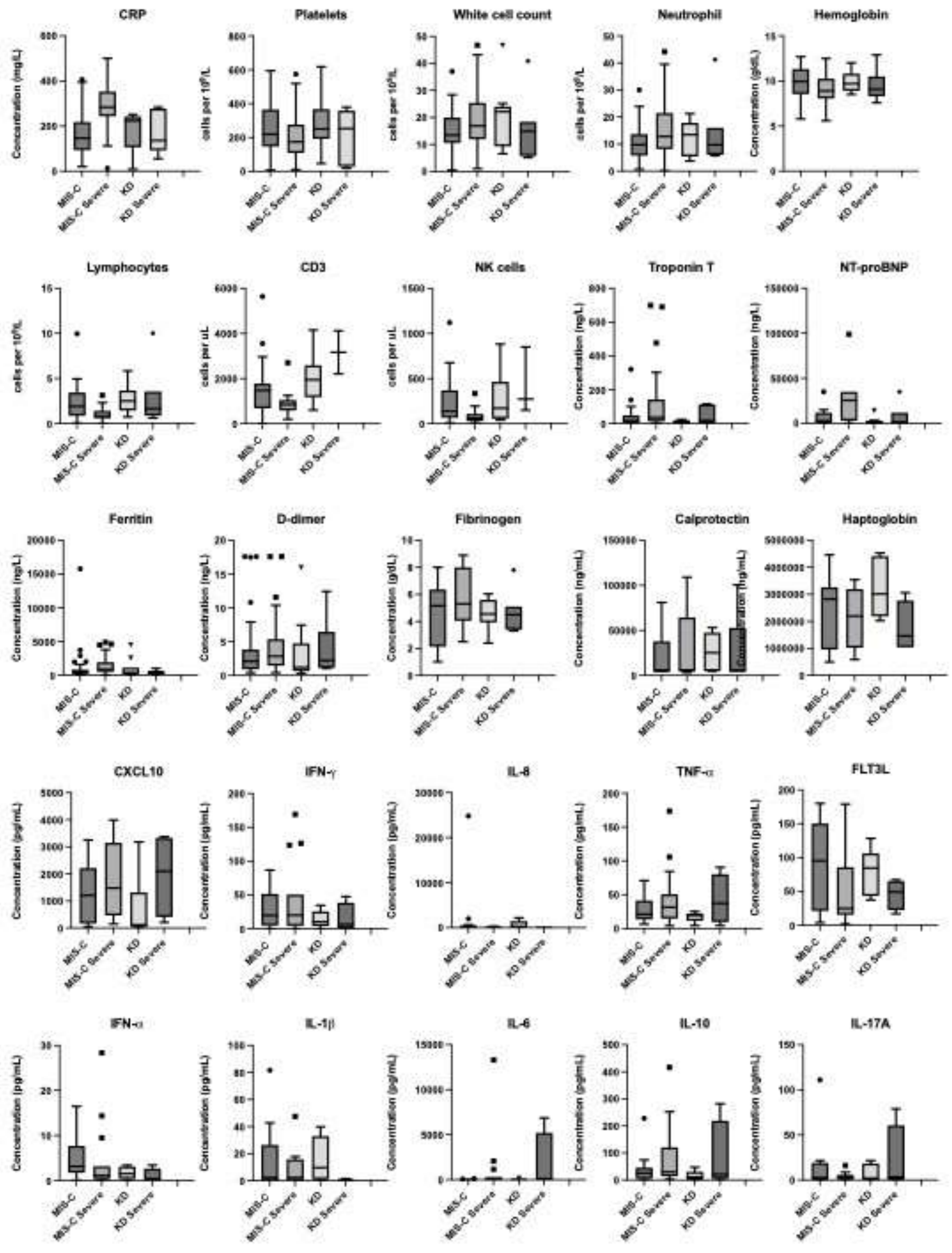

Supplement: Supplementary file 1 [file medi-104-e41516-s001.pdf]
